# The maternity care experiences of women living in a diverse UK urban city: a survey study

**DOI:** 10.1186/s12884-025-07773-z

**Published:** 2025-07-10

**Authors:** Kylie Watson, Charlotte Barber, Kimberley Farrant, Bethan McEvoy, Tracey Mills, Dame Tina Lavender

**Affiliations:** 1 Saint Mary’s Hospital, Manchester University NHS Foundation Trust, 5th Floor Saint Mary’s Hospital, Oxford Road, Manchester, M13 9WL UK; 2 Division of Nursing, Midwifery and Social Work, School of Health Sciences, Faculty of Biology, Medicine and Health, University of Manchester, Manchester, UK; 3 Centre for Childbirth, Women’s and Newborn Health, Liverpool School of Tropical Medicine, Liverpool, L3 5QA UK

**Keywords:** Inequalities, Equity, Maternity, Ethnic minorities, Experience survey

## Abstract

**Background:**

This study was undertaken to further understand the maternity experiences of women living in areas of high ethnic diversity and social deprivation. An anonymous self-reported on-line survey incorporating demographic, clinical outcome and validated tool (Experience of Maternity Care) questions, plus free text responses was used. Postnatal women, living in eight postcodes identified as areas of high socio-economic deprivation and ethnic diversity from a large urban NHS Trust in Northwest England were asked to complete the survey. Quantitative data was collected and analysed using appropriate statistics and free text responses were coded and thematically analysed.

**Results:**

361 women were included in the survey analysis. 74% of the sample identified as being from an ethnic minority. Black women (26/79, 33%) were more likely (*p* = 0.018) to attend their first antenatal visit at 12 or more weeks’ gestation compared to White (18/93, 19%) or Asian women (23/157, 15%). Black (*p* = 0.045) and multiparous (*p* = 0.025) women were more likely to report a positive postnatal experience and women who had been in the UK less than one year reported a more positive antenatal experience (*p* = 0.027). Most (82%) respondents did not mind being cared for during labour and birth by midwives or doctors they had not met before. Themes from free text responses included continuity of care, respectful care, communication, early labour care and access to timely pain relief.

**Conclusions:**

Using a targeted approach ensured an ethnically diverse sample and despite overall positive experiences of maternity care, negative experiences reflect similar themes previously identified within UK maternity care.

## Background

In the United Kingdom (UK) maternal mortality is highest amongst women from ethnic minority* (EM) backgrounds and living in deprived areas [1]. Ethnicity and socioeconomic status are both independently associated with increased risk of mortality, but are known to be highly correlated, with women from ethnic minorities more likely to live in socially deprived areas [2].

The reasons for continued inequalities are complex and multifaceted. Associated factors may include physiological influences, deprivation, late antenatal booking, poor clinical care, barriers to access, delay in seeking care, delay in prompt referral, language and translation barriers, and stereotyped expectations and understanding from healthcare staff [3–6]. Studies of the experiences of women from minority ethnic groups have found that compared to white women, they have more worries and fears about labour and birth [6] are more likely to feel disrespected, talked to in a way that they could not understand and have their choices denied [7]. A recent evaluation [8] of EM women’s experiences of maternity services found indications of mistreatment and poor care. Women-centred care (where there is engagement with the complexity of women’s lives as opposed to task oriented care) was an exception. Similarly, national survey data found women of lower socio-economic status were more likely to report that they were not treated respectfully or spoken to in a way they could understand by doctors and midwives [9]. Three recent reports [10–12] identified similar concerning themes. Cultural and religious competency amongst health workers has also been identified as an area for improvement to maximise women’s engagement and provide optimal care [13]. Where positive experiences were reported they are often associated with good communication, respectful and compassionate care, and opportunities to build trusting relationships with staff [8, 10, 11]. These disparities are a public health issue requiring the development of long-term strategies including involvement from diverse populations [14] and co-production of pathways.

Understanding women’s perceptions of maternity services is an important way of monitoring quality-of-care provision [15]. Within UK maternity services this is currently assessed by several different measures such as the Care Quality Commission national maternity survey [16] local ‘Friends and Family Test’ responses, complaints, compliments and listening events with organisations such as Maternity and Neonatal Voices Partnerships (MNVPs). To provide culturally competent services and tackle healthcare inequity, listening to feedback from EM groups, as well as those living in areas of social deprivation, is vital [17, 18]. This paper reports the first phase of a project which aimed to understand the maternity experiences of women living in areas of high ethnic diversity and social deprivation, classified by postcode, who received maternity care in a large urban tertiary NHS Trust in Northwest England.

*Within the context of this paper we use the term ethnic minorities to refer to all ethnic groups except White British and ‘other white’ minority groups.

## Methods

### Setting and sample

The study was conducted with women receiving care from a large NHS Trust where approximately 36% identify as EM background and 38% of residents live in the most deprived quintile [19]. An anonymous online survey was developed, incorporating demographic and clinical questions, an Experience of Maternity Care (EMC) tool [15] and free text boxes. The EMC tool was developed to examine salient aspects of experience retrospectively related to pregnancy, labour and birth, and the early postnatal period and validated using data from a national survey of women who had recently given birth in the UK [15]. The EMC tool [15] contained a total of 36 Likert-style questions, from strongly agree (score of 5) to strongly disagree (score of 1). To detect a standardised effect size of 0.5 between subscales within the EMC tool, with 80% power at 5% significance, it was calculated that at least 64 participants would be needed within each main ethnic group (using ONS classification). A total sample size of 350–400 was planned to ensure there were enough participants in each group.

### Recruitment, inclusion criteria and ethical considerations

Eligible participants were women living in eight postcodes identified as being in the lowest two Indices of Multiple Deprivation deciles and with high EM populations (identified from census data), who had given birth at the Trust within the last 10 weeks and were age ≥ 16. Recruitment took place from December 2020-February 2022, with a pause of 4 months due to the coronavirus pandemic. Flyers and posters (in English, Arabic, Urdu and Somali) with a link to the survey were placed in hospital and community settings for women to view and maternity staff were informed about the survey to signpost women. Copies of the flyer were given to women on discharge from the maternity unit. Study information was also shared across the Trust social media platforms, and community networks including the Maternity Voice Partnerships. Women were also directly recruited by a member of the research team from postnatal wards, community, and hospital postnatal clinics and support given to complete the survey if required using hospital interpreting services. Patient and Public involvement included meetings with local charity/community networks to gain feedback on the study recruitment flyer. An introductory cover page on the survey gave details of the purpose of the study, how data would be collected and stored and confirmation that participation was voluntary. Completion and submission of the survey indicated consent to take part. Ethical approval was obtained from the Bradford Leeds Research Ethics Committee (20/YH/0234) and undertaken in accordance with the Declaration of Helsinki prinicples.

### Data analysis

A secure web-based software platform (REDCap) was used to support data capture. Demographic and clinical outcome data were analysed descriptively and Chi-sqaure test for association used to determine significant differences where appropriate. For the EMC data analysis, ethnic groups were aggregated into Black/African/ Caribbean/Black British; Asian/Asian British, and White. An individual overall score (out of 60) was given for experiences in each of the three maternity care domains with higher scores indicating a more positive experience. Negative worded statements were reversed scored. For individual question analysis, ‘strongly agree’ and ‘agree to some extent’, and ‘strongly disagree’ and ‘disagree to some extent’ were combined to enable larger samples for analysis of women who either agreed or disagreed overall. Free text responses were coded as positive, negative or mixed, by two members of the research team (CB and KW), and thematically analysed.

## Results

### Demographics and clinical outcomes

In total 385 women started the survey, representing 11% of overall births from the 8 eligible postcodes during the recruitment timeframe. Of these, 24 participants provided only their age, baby’s age and first three letters of their postcode and were excluded from all analysis. A further 16 participants provided some additional demographic data only and did not commence the EMC questions; a total of 361 women were therefore included in the demographic and clinical outcome analysis. A total of 346 participants provided all demographic data and commenced the EMC questions (of which 295/346 completed all EMC questions) and were included in the EMC analysis. In total 295/346 (85%) completed all of the 36 EMC questions; data for statistical analysis was included if all 12 questions of the individual sections were completed. There were no obvious differences in those completing and not completing the survey. Table 1 shows demographic and clinical outcome data by aggregated ethnic groups. There were significant differences between the groups for age, being born in the UK, language spoken, length of time in the UK, being an asylum seeker or refugee, and religion. For clinical outcome data the only significant difference was Black women being more likely to book at 12 weeks or more of pregnancy than women from other aggregated groups and differences in type of infant feeding with low numbers of Black women formula feeding and more Asian women mixed feeding.

**Table 1:** Demographic and birth outcome data by ethnicity

	Demographics and birth outcomes by ethnicity	Asian/Asian British	Black African/ Caribbean/ black British	Mixed/multiple ethnic groups	Other ethnic group	White	total	*p* value
Totals		157 (43.5%)	79 (22%)	12 (3%)	20 (5.5%)	93 (26%)	361 (100%)	
age	16–19	0 (0%)	0 (0%)	0 (0%)	1 (33%)	2 (67%)	3 (100%)	0.0166
	20–24	12 (29%)	10 (24%)	1 (2%)	1 (2%)	18 (43%)	42 (100%)	
	25–29	57 (50%)	29 (25%)	4 (3.5%)	7 (6%)	18 (15.5%)	115 (100%)	
	30–34	58 (47.5%)	28 (23%)	3 (3%)	7 (6%)	25 (20.5%)	121 (100%)	
	35–39	25 (38.5%)	9 (14%)	3 (4.5%)	1 (1.5%)	27 (41.5%)	65 (100%)	
	40+	6 (40%)	3 (20%)	1 (6.5%)	1 (6.5%)	4 (27%)	15 (100%)	
parity	0	66 (41%)	31 (19%)	4 (2.5%)	8 (5%)	52 (32.5%)	161 (100%)	0.425
	1 or 2	70 (45%)	37 (24%)	7 (4.5%)	7 (4.5%)	35 (22%)	156 (100%)	
	3+	22 (50%)	11 (25%)	1 (2%)	3 (7%)	7 (16%)	44 (100%)	
Born in UK	Yes	52 (34.5%)	14 (9%)	9 (6%)	1 (0.5%)	75 (50%)	151 (100%)	< 0.005
	No	105 (50%)	65 (31%)	3 (1.5%)	19 (9%)	18 (8.5%)	210 (100%)	
Length of time in the UK	Less than one year	5 (33%)	6 (40%)	0 (0%)	1 (7%)	3 (20%)	15 (100%)	0.042
	1–5 years	51 (63%)	18 (22%)	0 (0%)	6 (7.5%)	6 (7.5%)	81 (100%)	
	6–10 years	15 (39.5%)	11 (29%)	1 (2.5%)	8 (21%)	3 (8%)	38 (100%)	
	More than 10 years	34 (45%)	30 (39.5%)	2 (2.5%)	4 (5%)	6 (8%)	76 (100%)	
Asylum seeker or refugee	Yes	3 (15%)	13 (65%)	0 (0%)	4 (20%)	0 (0%)	20 (100%)	< 0.005
	No	154 (45%)	66 (19%)	12 (3.5%)	16 (5%)	93 (27.5%)	341 (100%)	
Main Language	English	66 (34.5%)	35 (18%)	10 (5%)	3 (1.5%)	78 (41%)	192 (100%)	< 0.005
	Urdu	43 (100%)	0 (0%)	0 (0%)	0 (0%)	0 (0%)	43 (100%)	
	Arabic	1 (5%)	9 (45%)	0 (0%)	9 (45%)	1 (5%)	20 (100%)	
	English plus another language	12 (75%)	4 (34.5%)	0 (0%)	0 (0%)	0 (0%)	16 (100%)	
	Other	35 (39%)	31 (34%)	2 (2.5%)	8 (9%)	14 (15.5%)	90 (100%)	
Religion	Muslim	129 (70%)	30 (16%)	3 (2%)	16 (9%)	6 (3%)	184 (100%)	< 0.005
	Christian	15 (15%)	44 (45%)	4 (4%)	2 (2%)	33 (34%)	98 (100%)	
	No	3 (7%)	1 (2.5%)	4 (10%)	1 (2.5%)	33 (78%)	42 (100%)	
	Other	10 (27%)	4 (11%)	1 (2.5%)	1 (2.5%)	21 (57%)	37 (100%)	
Education	None	13 (41%)	9 (28%)	1 (3%)	3 (9%)	6 (19%)	32 (100%)	0.424
	GCSE or equivalent	27 (43%)	10 (16%)	3 (4.5%)	4 (6.5%)	19 (30%)	63 (100%)	
	A level or equivalent	19 (51.5%)	9 (24.5%)	1 (2.5%)	2 (5%)	6 (16.5%)	37 (100%)	
	Diploma or equivalent	14 (32%)	17 (39%)	1 (2%)	3 (6.5%)	9 (20.5%)	44 (100%)	
	Undergraduate Degree or higher	83 (45%)	33 (18%)	6 (3%)	8 (4%)	53 (30%)	183 (100%)	
	missing	1 (50%)	1 (50%)	0 (0%)	0 (0%)	0 (0%)	2 (100%)	
First health professional seen in pregnancy	Midwife	117 (43%)	56 (20.5%)	11 (4%)	12 (4.5%)	77 (28%)	273 (100%)	0.051
	GP	39 (52%)	18 (24%)	1 (1.5%)	5 (6.5%)	12 (16%)	75 (100%)	
	Other/missing	1 (7.5%)	5 (38.5%)	0 (0%)	3 (23%)	4 (31%)	13 (100%)	
Gestation at booking	Less than 12 weeks	126 (48%)	44 (17%)	9 (3.5%)	14 (5%)	69 (26.5%)	262 (100%)	0.0177
	12 weeks or more	23 (30.5%)	26 (35%)	2 (2.5%)	6 (8%)	18 (24%)	75 (100%)	
	Don’t know/missing	8 (33.5%)	9 (37.5)	1 (4%)	0 (0%)	6 (25%)	24 (100%)	
Gestation at birth	37 weeks or more	139 (43%)	73 (22.5%)	11 (3.5%)	17 (5%)	85 (26%)	325 (100%)	0.686
	Less than 37 weeks	15 (53.5%)	3 (10.5%)	1 (3.5%)	3 (11%)	6 (21.5%)	28 (100%)	
	Missing	3 (37.5)	3 (37.5)	0 (0%)	0 (0%)	2 (25%)	8 (100%)	
Labour onset	Spontaneous	67 (43%)	33 (21%)	5 (3%)	11 (7%)	41 (26%)	157 (100%)	0.704
	Induction	58 (47%)	22 (18%)	3 (2.5%)	7 (5.5%)	33 (27%)	123 (100%)	
	Elective caesarean	29 (40%)	20 (28%)	4 (5.5%)	2 (2.5%)	17 (24%)	72 (100%)	
	Missing	3 (33.5%)	4 (44.5%)	0 (0%)	0 (0%)	2 (22%)	9 (100%)	
Type of birth	Spontaneous vaginal birth	81 (45%)	39 (21.5%)	4 (2%)	12 (6.5%)	45 (25%)	181 (100%)	0.519
	Assisted vaginal birth	17 (42.5%)	5 (12.5%)	0 (0%)	2 (5%)	16 (40%)	40 (100%)	
	Elective caesarean	25 (42.5%)	14 (24.5%)	4 (5.5%)	1 (7%)	15 (20.5%)	59 (100%)	
	Emergency caesarean	31 (42.5%)	18 (24.5%)	4 (5.5%)	5 (7%)	15 (20.5%)	73 (100%)	
	Missing	3 (37.5%)	3 (37.5%)	0 (0%)	0 (0%)	2 (25%)	8 (100%)	
Method of feeding when completing survey	Breastfeeding	41 (32%)	33 (26%)	3 (2%)	11 (9%)	40 (31%)	128 (100%)	< 0.005
	Formula	34 (48.5%)	5 (7%)	4 (5.5%)	0 (0%)	27 (39%)	70 (100%)	
	Mixed	78 (50.5%)	38 (24.5%)	5 (3%)	9 (6%)	24 (16%)	154 (100%)	
	Missing	4 (44.5%)	3 (33.5%)	0 (0%)	0 (0%)	2 (22%)	9 (100%)	

### Experience of maternity care scores

Scores were calculated for each subscale (antenatal, labour and birth, and postnatal) of the EMC tool for three main aggregated groups (that all had more than 64 participants). The combined overall mean scores were similar for each subscale: antenatal 49.71/60 (SD 7.9), intrapartum 51.24/60 (SD 8.6) and postnatal 51.24/60 (SD 8.6; Table 3). Antenatal or intrapartum experience scores were not related to maternal ethnicity. Black women were more likely to report a positive postnatal experience (*p* = 0.045) than Asian or White women, and multiparous women were more likely to report a better postnatal experience than primiparous (*p* = 0.025). Women who had been in the UK for less than one year reported better antenatal scores than women being in the UK for differing times with the women being in the UK for between 6 and 10 years reporting the lowest scores for antenatal experience (*p* = 0.027). There were no differences between length of time in the UK and intrapartum or postnatal scores (see Table 2*EMC Scores table*).

**Table 2:** EMC scores table

	Overall mean (SD)	Asian/Asian British mean (SD)	Black/AFrican/Black CAribbean mean (SD)	White mean (SD)	*p* value	Primigravida mean (SD)	Multigravida mean (SD)	*p* value	UK less than a year	UK 1–5 years	UK 6–10 years	UK over 10 years	*p* value
**Antenatal score**	49.71 (7.88)	49.32 (8.7)	51.41 (7.15)	49.31 (7.36)	0.147	48.99 (SD 8.41)	50.64 (7.39)	0.07	55.33 (2.74)	50.17 (SD 8.31)	47.88 (SD 7.94)	51.76 (SD 7.25)	0.027
**Intrapartum score**	51.25 (8.6)	50.61 (9.28)	52.28 (7.28)	51.34 (9.4)	0.424	50.56 (SD 9.28)	51.89 (8.36)	0.197	54.58 (4.14)	50.32 (SD8.91)	52.22 (SD 7.92)	53.44 (SD 6.82)	0.086
**Postnatal score**	51.24 (8.6)	51.1 (8.69)	53.18 (8.3)	49.55 (9.28)	0.045	49.68 (SD 9.51)	52.15 (SD 8.03)	0.025	56.45 (3.39)	52.43 (7.74)	52.82 (SD 5.39)	52.30 (SD 8.08)	0.378

### Individual question responses

Most women were positive about the overall care they received during pregnancy (311/345, 90%) labour and birth (291/333, 87%) and postnatally (263/295, 89%)– see Table 3 Individual question analysis. Within the antenatal period 93% (324/346) of women felt they had the ‘right number’ of antenatal contacts with a midwife or doctor and 89% (308/346) were ‘happy with the number of health professionals who cared for me’ but only 55% (189/347) of women stated that they received continuity of carer during their antenatal care. One fifth of women (74/346,21%) felt that health professionals did not always talk to them in a way that they could understand but 90% (313/346) of women felt that they were given all the information they needed in the antenatal period. During labour, 25% (83/334) of respondents felt that they needed more support, 19% (63/333) did not feel that their needs for pain relief were fully met and 25% (84/333) of women felt that ‘staff could have done more to make me feel more in control’. However, communication, being treated as an individual, feeling safe and having confidence in staff all scored highly (> 85%). 82% (272/333) of women ‘did not mind being looked after in labour by someone I had not met before’. During the postnatal period 71% (222/313) felt that they had stayed long enough in the hospital after the birth.

**Table 3:** Individual question analysis

Survey statement	Agree (strongly agree and agree to some extent)	Neither agree nor disagree	Disagree (strongly disagree and disagree to some extent)	Total
**Antenatal - combined overall mean score 49.81/60 (SD 8.6)**				
A1 I felt I had the right number of antenatal checks with the midwife/doctor	324 (93%)	10 (2.8%)	12 (3.5%)	346
A2 My care provider(s) gave me all the information I needed	313 (90.4%)	13 (3.7%)	20 (5.8%)	346
A3 I always saw the same midwife/doctor for my antenatal checks	189 (54.5%)	24 (6.9%)	133 (38.3%)	346
A4 Health professionals did not always talk to me in a way I could understand	74 (21.3%)	19 (5.5%)	253 73.1%)	346
A5 Antenatal appointments were too short to discuss any concerns about my pregnancy	61 (17.6%)	37 (10.7%)	248 (71.7%)	346
A6 I was not involved enough in decisions about my antenatal care	39 (11.3%)	29 (8.4%)	278 (80.3%)	346
A7 I was happy with the number of health professionals who cared for me during my pregnancy	308 (89.0%)	19 (5.5%)	19 (5.5%)	346
A8 I was not given enough explanations about antenatal scans and tests	45 (13.0%)	21 (6.1%)	279 (85.5%)	345
A9 I was not given enough information to make decisions about my antenatal care	37 (10.7%)	32 (9.2%)	277 (80.1%)	346
A10 I would have liked more antenatal checks and scans	106 (30.6%)	51 (14.7%)	189 (54.6%)	346
A11 During pregnancy, I did not feel well cared for by health professionals	30 (8.7%)	20 (5.8%)	296 85.5%)	346
A12 Overall, I was very pleased with the care I received in pregnancy	311 (90.1%)	11 (3.2%)	23 (6.7%)	345
**Intrapartum combined overall mean score 51.21/60 (SD 8.6)**				
B1 Staff communicated well with me during labour and birth	307 (91.6%)	8 (2.4%)	20 (6.0%)	335
B2 I needed more staff support during labour and birth	83 (24.9%)	35 (10.5%)	216 (68.7%)	334
B3 Everything was explained to me well during labour and birth	298 (89.2%)	11 (3.3%)	25 (7.5%)	334
B4 I was treated as an individual by staff	285 (85.3%)	21 (6.3%)	28 8.4%)	334
B5 I was not involved enough in decisions about procedures that were carried out (e.g. breaking waters, caesarean section)	42 (12.6%)	26 (7.8%)	266 (79.6%)	334
B6 Health professionals left me alone more than I would have liked	54 (16.2%)	34 (10.2%)	245 (73.6%)	333
B7 I felt that my pain relief needs were not managed well	63 (18.9%)	22 (6.6%)	248 (74.5%)	333
B8 I felt safe in the labour and birth environment	296 (88.9%)	18 (5.4%)	19 (5.7%)	333
B9 The staff could have done more to help me to feel in control of my labour and birth	84 (25.2%)	31 (9.3%)	218 (65.5%)	333
B10 I had confidence and trust in the staff caring for me	299 (89.8%)	20 (6.0%)	14 (4.2%)	333
B11 I did not mind being looked after by midwives or doctors I had not met before	274 (82.3%)	21 (6.3%)	38 (11.4%)	333
B12 I had the best possible care during labour and birth	291 (87.4%)	22 (6.6%)	20 (6.0%)	333
**Postnatal combined overall mean score 51.13/60 (SD 8.7)**				
C1 I received enough care and attention from staff on the postnatal ward	273 (86.7%)	9 (2.9%)	33 (10.5%)	315
C2 I stayed in hospital as long as I wanted after the birth	222 (70.9%)	37 (11.8%)	54 (17.3%)	313
C3 I was treated as an individual by midwives/doctors after the birth	263 (84.6%)	22 (7.1%)	26 (8.4%)	311
C4 After I had given birth, health professionals treated me as though I was no longer important	49 (15.8%)	25 (8.1%)	236 (76.1%)	310
C5 I had enough information from health professionals about how to care for my baby	255 (82.3%)	22 (7.1%)	33 (10.6%)	310
C6 I was able to build a good relationship with the healthcare professionals I saw after coming home	220 (74.3%)	47 (15.9%)	29 9.8%)	296
C7 I was not given the advice and information I needed by health professionals after my baby was born	34 (11.5%)	25 (8.4%)	237 (80.1%)	296
C8 There was not enough time to talk over my concerns with health professionals	46 (15.6%)	24 (8.1%)	225 (76.3%)	295
C9 I had all the checks I needed after the birth	259 (87.5%)	17 (5.7%)	20 (6.8%)	296
C10 After the birth of my baby, I knew who to contact if I had questions or concerns	260 (87.8%)	19 (6.4%)	17 (5.7%)	296
C11 The postnatal care I received did not meet the needs of me and my baby	34 (11.5%)	28 (9.5%)	233 (79.0%)	295
C12 Overall I was very pleased with the quality of my postnatal care	263 (89.2%)	21 (7.1%)	11 (3.7%)	295

### Free text responses

**Fig. 1:**
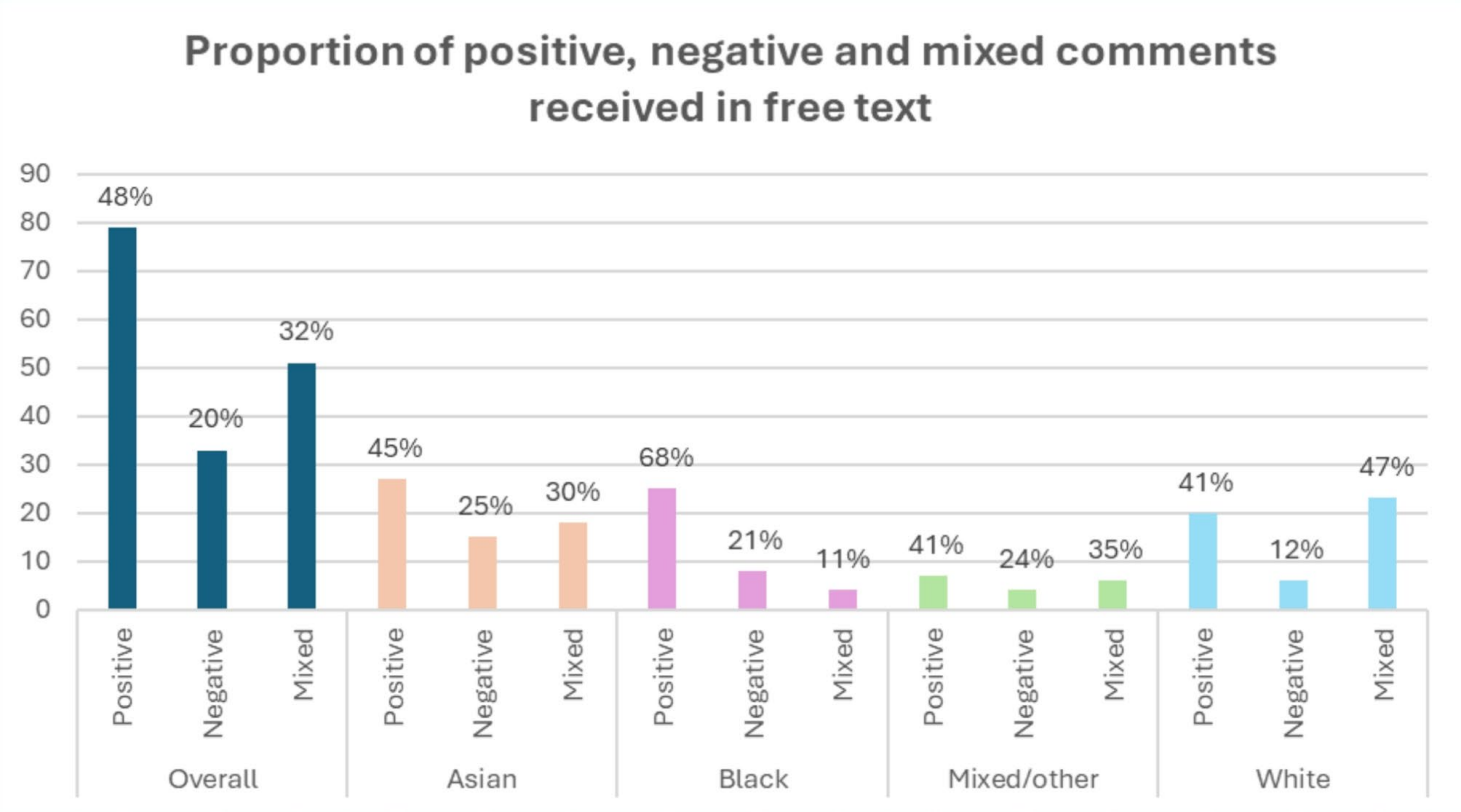
Breakdown of free text comments into category by ethnicity

In total 163 free text comments (42% of respondents) were recorded. Overall, 48% of comments were positive, 32% mixed and 20% negative. Figure 1 (*Breakdown of free text comments into category by ethnicity*) shows the breakdown of positive, negative, and mixed comments overall and by ethnicity. Analysis found that there was a significant difference in the distribution of response types (*p* < 0.005) across the four different ethnic groupings with less mixed responses and more positive responses for Black /African/Black Caribbean than other groups.

### Free text responses

Where women described positive episodes of care, they reported ‘feeling safe’, listened to, respected, and supported to make fully informed decisions. Women used positive words and phrases to describe care that had a lasting impact, expressed their appreciation for the caring and attentive staff and a widespread gratitude for the NHS.

*Excellent service*,* I really appreciated the fact that all professionals listened to me rather than advising what I needed to do after birth. I felt my opinions mattered and that both my physical and mental health needs were considered (Asian British woman).*

*I felt safe and prioritised the whole way through. (White woman)*

Several comments highlighted the importance of clear communication: respondents were pleased that midwives and doctors took the time to explain procedures, answer questions, and ensure that they were well-informed about their care.

*It was an amazing experience staying here. At the start of my stay at the hospital it was extremely painful but the support that I received really helped me. Doctors*,* midwives*,* and every team member made me feel very important and treated me as an individual. My concerns regarding me and my baby’s health were clearly and lovingly answered and handled*,* everyone was so supportive. (Asian woman)*

Continuity of carer (CoC, where midwives are organised into small teams and each midwife aims to provide antenatal, intrapartum, and postnatal midwifery care with support from the wider team for afterhours care) was a theme within positive, negative, and mixed comments. Comments reflected the value women attached to relational care, particularly being able to see the same midwife throughout their antenatal journey. Some women were disappointed they did not have the opportunity to see their antenatal midwife in the postnatal period.

*I had continuity and my midwife was present at my birth. This was good as she knew me and followed me*,* so I had better care this time. She had all the right information about me. (Black Caribbean woman).*

*My community midwife was excellent. I was disappointed that I didn’t see her again after I gave birth*,* would have been nice to have end-to-end care (mixed ethnic group woman).*

*We would have liked to have more consistency of continuity of midwives during the pregnancy as it seemed to change every appointment. (White woman).*

Negative comments highlighted a range of issues and concerns, with several recurring themes. Many comments reflected frustration with inadequate communication, particularly regarding ultrasound scans and the decisions surrounding induction of labour. Some women felt that they were not adequately informed about their pregnancy and care plans, leading to confusion and anxiety. Lack of information and guidance on postnatal care, infant care, and what to expect during the hospital stay were common concerns. First-time mothers felt unprepared and vulnerable. Many women left free text comments about delay in the diagnosis of labour. This included women on the induction of labour (IOL) pathway and women attending triage in early labour.

*We had been in triage from 5-11pm and then were sent home. We came back an hour later and I had the baby 20 min later on triage. We struggled to get back to the hospital*,* all the taxis were busy*,* we shouldn’t have been sent home (Asian British woman).*

*I laboured at home and rang triage a few times. As a first-time mum I would have preferred to have been taken more seriously when it came to pain; mostly because I was told if I could speak the pain wasn’t that bad (Black African woman).*

These comments also tied in with the theme of women receiving timely and effective pain management, both in labour and on the postnatal ward.

*I was a first-time mum and in so much pain and this midwife didn’t help when I asked for pain relief. It turns out that I was 6 cm dilated by the time she examined me*,* she completely ignored me*,* and I had struggled managing the pain having got to 6 cm (Asian British woman).*

*I really felt like I wasn’t listened to and that my pain was ignored and dismissed. I was begging for care and to be seen but kept being told to wait whilst I was in agony (Asian woman).*

Issues with care on the postnatal ward was also a recurrent theme within the mixed and negative comments, largely related to a lack of privacy in shared bays, delay in receiving assistance from staff and not all staff being friendly or approachable.

*One particular midwife did not come when I called for assistance via the buzzer and my husband had to go and find her. She made me feel as though I was being dramatic. (Black British woman)*

*There were issues on the postnatal ward during the night. I had to ring the bell for a long time to get care. (Asian British woman).*

## Discussion

### Main findings

Using a targeted approach, focusing recruitment of women living in certain postcodes, has ensured that the experiences and voices of an ethnically diverse group of women living in areas of deprivation were heard in the study. The combined EMC experiences for the sample were overall positive and there was little difference between antenatal, intrapartum and postpartum aspects of maternity care. However, there were some outliers when looking at individual questions within the three domains. Black women and multiparous women were more likely to report better postnatal experiences and women living in the UK for less than one year reported more positive antenatal experiences. Within the demographic data the clinical differences between the groups were that Black women were more likely to book later than 12 weeks of pregnancy and lower numbers of Black women formula feeding and more Asian women mixed feeding. There was a spread of positive, negative, and mixed comments which provided further insight and contextual vignettes about the experiences of maternity care for this sample. Women reported examples of excellent personalised care, where they felt well listened to and safe. Women appreciated CoC when they received it, with others wishing they could have had better CoC. Negative themes related to long waiting times, delayed diagnosis of labour, and delayed, or timely access to, pain relief.

### Strengths and limitations

A strength of the study was the diverse sample of women, who historically have been labelled as a ‘hard to reach’ group for research [20]. The research aimed to recruit 350–400 participants, however only 346 participants were included in the EMC analysis due to incomplete data. Whilst only representing 11% of births within targeted postcodes the sample provided rich quantitative and qualitative data. The research team recognise that this may have been influenced by the use of a questionnaire that required a good understanding of English. Due to funding constraints, we were unable to provide translations in multiple languages. We had planned to involve women identified as ‘Cultural Connectors’ from a local VCSE organisation to help engage with participants who did not speak English as a first language. However, restrictions during the COVID-19 pandemic prevented this, limiting our ability to reach non-English-speaking women. Although women who did not speak English were included through use of hospital interpretation services, use of cultural connectors may have also supported data completeness for those with English as an additional language, or experiencing cultural barriers.

Whilst the use of a survey does not always provide rich explanatory data, using both a quantitative tool and free text responses allowed triangulation with free text responses contextualising some of the Likert question responses. There were no specific questions relating to cultural considerations (within the EMC or as stand-alone questions) however this has been explored in a follow-up qualitative phase. A large proportion of participants were educated to a degree level or higher, and this overrepresentation is common in other similar studies [11]. This may reflect greater familiarity with research and a higher willingness to participate. This also highlights a limitation in the targeted postcode approach as whilst being identified as living in a deprived postcode, many women in the sample were also highly educated. The authors acknowledge grouping by ethnicity into broad groups implies homogeneity and does not allow for the diversity of cultures within the group to be represented. For example, black women from the Caribbean may have very different expectations and experiences than African women However, it was a useful way to explore the data to begin to investigate local experiences and target future work.

The research team for this study are all white, professional women which may also have had some influence on recruitment and responses. Acknowledging this limitation, the research team engaged in with training around cultural safety and adopted a reflexive approach throughout the study. This was underpinned by cultural humility, an ongoing process of self-reflection and learning.

### Interpretation

Black women were more likely to book for antenatal care after 12 weeks of pregnancy, as reflected in other literature [21, 22]. Late attendance is known to be associated with poor obstetric and neonatal outcomes amongst minority ethnic women with some suggested reasons for this being cultural prohibitions surrounding early disclosure of pregnancy and concern around immigration issues [23]. More broadly there is evidence that Black and other EM women can experience distrust of healthcare professionals leading to a reluctance to engage and attend services [14]. More evidence is needed to develop specific interventions within local populations to increase earlier booking rates among Black women and this is a focus of work in the local system. Given reports of poor care experienced by women from ethnic minorities we were somewhat surprised at our overall findings of mostly positive experiences of care demonstrated by high combined mean scores using the EMC tool. It is not entirely surprising that multiparous women reported better experiences of postnatal care due to having navigated early parenthood previously, or this may be due to care that surpassed low expectation, but the similar finding for Black women needs to be explored further and may need to be treated with caution. Similarly, the higher antenatal scores for women being in the UK less than a year before completing the survey also warrants further exploration and may be due to differing expectations of care.

Within the individual question responses there were some areas that correlated with some of the free text responses. CoC was identified by some women as an important component of their care and has been shown to improve safety, clinical outcomes, and experience [24]. This model of care has received a large amount of attention since the publication of Better Births [15] with ambition on it being the default model of care for all women [25, 26]. Within this sample only 55% of respondents agreed or mostly agreed with the statement ‘I always saw the same midwife/doctor for my antenatal checks’. During the time that the survey was undertaken there were only two CoC midwifery teams providing care at the Trust and the response indicates that even within traditional antenatal care less than half of women are seeing the same midwife for antenatal care. Free text comments highlighted that for some women, seeing the same midwife during the pregnancy was hugely beneficial and women also wished to see this same midwife after the birth reflecting literature on relational care [27]. Only one woman commented on being cared for by a known midwife during labour, reflective of the low level of CoC models within the Trust. Of interest is that 82% somewhat or strongly agreed with the statement ‘I did not mind being looked after by midwives or doctors I had not met before’ during labour and birth. This is not reflective of findings from the National Maternity Review [28] where authors reported that they ‘found almost total unanimity from mothers that they want their midwife to be with them from the start, through pregnancy, birth and then after birth’ (p 4). Findings from our survey indicate that focusing on antenatal and postnatal continuity may have positive effects on experience. This is not to minimise the known benefits of full CoC, (from the antenatal period through to intrapartum and postnatal care), but where services are finding this challenging to implement then a focus on antenatal and postnatal continuity could have significant benefits for women and increased satisfaction for midwives [29, 30].

Almost 20% of the respondents in this survey reported their pain relief during labour was inadequate, supported by free text responses which also highlighted insufficient postnatal analgesia. This is reflective of literature highlighting inequalities between some groups in accessing pain relief during the perinatal period [11, 31, 32]. A number of black and Asian women in our survey also reported they were not listened to when seeking advice around symptoms of labour resulting in a subsequent delay in diagnosis of labour onset and timely and appropriate pain relief. This reinforces the call for women from ethnic minorities to be really heard, listened to, and believed to reduce inequalities and challenge racist assumptions and stereotypes around managing pain during the perinatal period [11]. A large amount of focused work has been undertaken within the Trust subsequent to the survey, demonstrating marked improvement in triage waiting times and care, pain relief availability in all areas and commitment to listening to all women.

## Conclusions

Whilst acknowledging literature on EM women’s, mostly poor, experiences of care we wished to understand more local-level data that reflected the demographics of women receiving maternity care. This detailed data will facilitate co-production of future work, including the development of an Appreciative Inquiry [33] qualitative phase focusing on what components contribute to excellent care for women from EM groups and living in deprived areas.

As experiences and outcomes vary for sub-groups within broad ethnic groups, future studies should breakdown data in more detail and acknowledge that experiences are not all similar within racialised women categorised into large groups. Identifying which groups are at risk of specific outcomes will enable the development of targeted interventions for different communities, rather than a ‘treat all’ approach for EM women. This could include working with health professionals and women to co-design interventions to maximise positive experiences for all and address the consistent negative themes that are being repeated across the literature. In-depth appreciative inquiry interviews informed by this work will be published separately.

## Data Availability

The datasets used and/or analysed during the current study are available from the corresponding author on reasonable request.
